# The Pitfalls of Utilizing “Goals of Care” as a Clinical Buzz Phrase: A Case Study and Proposed Solution

**DOI:** 10.1089/pmr.2020.0063

**Published:** 2020-10-06

**Authors:** Adrienne Klement, Sean Marks

**Affiliations:** Section of Palliative Care, Department of Medicine, Medical College of Wisconsin, Milwaukee, Wisconsin, USA.

**Keywords:** consultation, goal-concordant care, goals of care, standardization, unified definition

## Abstract

Assistance with discussing goals of care is one of the most common reasons clinicians seek out palliative care consultation. In practice though, the phrase “goals of care” is often utilized as a buzz phrase that lacks a shared understanding of its clinical relevance. We present a case example in which breakdowns in communication occurred between a patient and clinicians due to misunderstandings of the meaning of the phrase “goals of care.” Subsequently, we review the literature to propose a unified definition of “goals of care” in hopes to minimize differences in what this phrase implies in clinical practice. We also seek to introduce a standardized process for establishing goals of care that may offer a more reliable and measurable method to promote goal-concordant care.

## Introduction

Considering its emerging focus in quality and outcome measurements, clinicians are increasingly aspiring to deliver goal-concordant care for their seriously ill patients.^1,2^ Goal-concordant care has been referred in the published literature as clinical care that helps a patient reach an identifiable goal and respects any treatment preferences or limitations the patient has placed on his or her clinical care.^3^ Yet, reliably determining and measuring whether outcomes were concordant with patients' goals have proven difficult.^4^ Palliative care consultation teams are increasingly utilized to address unmet care needs of patients with life-limiting illnesses in the hopes of providing goal-concordant care more consistently.^5–7^ Establishing goals of care is one of the most frequent reasons referring clinicians seek out palliative care consultation.^8,9^ This trend is likely to continue, since establishing clear and medically appropriate goals of care by clinicians with specialized skills in serious illness communication has been shown to translate into higher value care.^10,11^

In this case analysis, we review how a lack of a unified definition of the phrase “goals of care” contributed to communication breakdowns in the care of a seriously ill patient for whom a referring provider sought out a “goals-of-care” palliative care consultation. We illustrate how various members of the clinical team did not share the same expectations of what a “goals-of-care” conversation entails, making it challenging to determine whether “goal-concordant care” was achieved through the consultative process. We utilize this case to then propose a clearer two-step process in addressing “goals-of-care” consultations, which we hope will foster better reliability in achieving goal-concordant care.

## Case Example

A palliative care consultation team receives a text page from a health unit communicator (HUC) eliciting a request for consultation from a hospitalist regarding a patient admitted with a malignant bowel obstruction and functional decline in the context of widely metastatic colorectal cancer. The HUC assigned to the patient's medical ward conveys that surgical management is no longer being considered for the patient, hence palliative care consultation is being solicited to help establish medically reasonable “goals of care.” Per the HUC, if further information is desired, the palliative care team should review the electronic medical records (EMRs) or page the hospitalist directly. Upon reviewing the EMRs, the palliative care clinician identifies discussions between the hospitalist and the patient about his global health picture, prognosis, and desire for information on next steps. An attempt is made to reach the hospitalist, but a covering provider returns the page, who does not have further details to offer. Considering institutional consult response standards, the consultant opts to complete the consult sans a direct conversation with the referring provider.

When asked to elicit his understanding of illness and care preferences, the patient conveys to the palliative care team his wish to avoid physical suffering and future hospitalizations knowing now that his cancer is not curable. The presence of widely metastatic cancer and declining performance status lead the palliative care team to assume that no further systemic cancer treatments are going to be offered. Through the natural course of their conversation with the patient, they recommend home hospice services for his posthospitalization care.

When this is reported back to the referring hospitalist, he conveys displeasure that hospice care was discussed, as he hoped the palliative care team would adhere to the consultation question: help with addressing “goals of care.” The hospitalist clarified that since the patient was naive to systemic cancer treatments, he was concerned that hospice care discussions would be premature and confuse the patient. By “goals-of-care discussions,” he clarifies that he meant eliciting patient care preferences for code status and whether transfer to the intensive care unit should be pursued if the patient became critically ill: “Since you are the experts in communication, I thought you could do this better than me.”

## Case Analysis

Numerous breakdowns in communication occurred between the referring providers and palliative care consult team in this case study. Perhaps foremost, the palliative care clinician did not insist on a direct conversation with the appropriate referring provider so that a more nuanced consult question could have been better articulated or negotiated in real time.^12^ The pace of inpatient care and the complexity of care teams involved contributed to the substandard interteam communication and likely compelled both teams to pursue shortcuts with the consultation intake process.^12^ Another structural issue was a lack of a clear understanding of the phrase “goals of care.” Without a clear operational consensus on the meaning of the phrase within the medical community, “goals of care” often is used as a vaguely defined “buzz phrase” to connote that a patient is not doing well clinically. Although one clinician may employ the phrase to connote that code status needs to be addressed, another may interpret it to mean that employing life prolongation as the fundamental goal of medical interventions needs to be reconsidered.

Bern-Klug argued that the implicit goal of medical care is cure or life prolongation, and when either of these aims is no longer possible, more reasonable medical goals of care are often not explicitly stated by clinicians.^13^ Weissman and Meier defined “goals of care” as physical, social, spiritual, or other patient-centered goals that arise after an informed discussion of the disease(s), prognosis, and treatment options.^14^ Stone offered a list of over nine potential goals of care for medical treatment including avoidance of premature death, optimized quality of life, or relief of suffering.^15^ Stanek utilized a concept clarification process in hopes of assimilating these disparate definitions in the literature. Through this methodology, she defined “goals of care” as desired health expectations formulated through thoughtful interactions between the health care team and a human being.^16^

Although Stanek's definition offers a potentially unified concept for “goals of care,” in our experience, there still is not a uniformly accepted definition that is applied reliably throughout clinical situations, nor a standardized process for establishing goals of care, which are essential in the current culture of inpatient practice involving multiple clinicians and care teams. Although having a standardized and uniformly accepted definition for “goals of care” certainly would not replace the need for direct communication between referring and consulting providers, we do believe it could potentially reduce communication misadventures between care services, or even with patients, knowing that time pressures to complete consultations efficiently and communication challenges in dealing with increasingly complex care teams are likely to continue in inpatient care environments.

## Proposed Definition for Establishing Goals of Care

To stratify communication between interdisciplinary teams, patients, and surrogates, we propose a merger of commonly referenced goals-of-care definitions proffered into a clinically applicable two-step “goals-of-care” process that we believe could be standardized throughout health care systems.

Step 1 of this process (Table 1) involves identifying whether the primary intent of medical interventions should be, in broad terms, to cure, to prolong life, or to focus on comfort. By labeling medical goals of care in a somewhat reductive, yet tangible manner, we hope to foster more successful protocols and standardization of the process of establishing goals of care.

**Table 1.** The First Step: Ascertain Whether There Is a Shared Understanding of the Primary Aim of Medical Treatment

Cure disease
Prolong life through control of disease and/or rehabilitation
Maximize comfort-oriented care

For proposed medical interventions, there is an expected “top level” treatment goal to cure. If cure is not possible or desired, disease management, life prolongation, physical strengthening, or rehabilitation may be acceptable. If these intents are not achievable or desired, comfort or the relief of suffering with death expected is an acceptable goal.

Step 2 of the process references recommended practices by major medical groups, such as the age-friendly health systems (AFHS) initiative,^17^ and is described in Table 2. This part of the process would likely be more difficult to standardize, as they would fundamentally be individualized by patient values and the underlying medical realities. Although there are multiple published peer-reviewed resources to help providers perform value finding with patients, we must consider how challenging it can be for any person to articulate fundamental and sustained care values and preferences that drive decision making. Establishing more nuanced patient-centered goals of care becomes even more challenging if there is not a shared understanding among patients and clinicians of the primary aim of medical interventions. Hence, this process is often an iterative and artful one between provider and patient, which involves assessing the patient's hopes, fears, worries, and sense of purpose through illness.^18–20^ It is best accomplished after step 1, and may require help of an interdisciplinary team of palliative care specialists.^21^

**Table 2.** The Second Step: Elicit Patient-Centered Goals of Medical Interventions

Goal	Example
Functional	Improve or maintain current functional status or mobility
Survival	Avoid premature death, maximize dignity, and/or quality of life
Family	Attend an event, leave a legacy, avoid burden on family
Mentation	Maintain cognitive status or maximize alertness
Psychosocial	Make peace with family/faith, complete a will

This table depicts types of patient-centered goals and gives examples of each. Once patients, surrogates, and treating clinicians are aligned and have a shared articulation of the first step (Table 1), then more nuanced patient-centered goals of care can be achieved.

## Practical Application

Resources and guides are available to help clinicians improve their skills in leading “goals of care” or serious illness discussions.^17,22,23^ These tools have been successful because they incorporate agreed-upon communication principles such as prognostic disclosure, patient values, and care preferences into the shared decision-making process.^24^ Despite the availability of these resources and educational platforms, clinicians continue to seek help from palliative care specialists for goals-of-care discussions. As exemplified in our case, the “goals-of-care” process employed by palliative care specialists may seem variable or even like a “black box” to referring physicians, as exemplified by the hospitalist in our case envisioning the palliative care consultative team as the “communication experts” who would be better adept at addressing code status preferences.

We worry that as a specialty, we may feed into this mystery of what we do in our consultative work by promoting ourselves as communication experts for patients with serious illness. As opposed to promoting our communication skills as expert-like, we should be striving to define our processes so that it can be more easily reproduced by clinicians across specialties and institutions. This should help referring teams anticipate what a palliative care consultation will entail through a transparent disclosure of the consultative process they will be likely to employ upon the initial visit. Specifically, that they could first assess the patient's understanding of the primary intent of medical interventions by using step 1 (Table 1). If perceived intents do not match up, referring providers would have a clearer indication of when a formal “goals of care” or serious illness discussion would be needed to negotiate consensus. By articulating this aspect of the consultative process so plaintively to referring teams, we hope that more clinicians will perceive step 1 in our process as an achievable and fundamental task in the care of the seriously ill. Perhaps this will lead clinicians to offer more cogent clinical recommendations within the framework of an overarching goal of medical care, rather than piecemeal discussions of various treatment options (e.g., only addressing code status) that usually result in imprudent medical plans.

We also imagine better utilization of consultative policies embedded in the EMRs to achieve this goal. The AFHS offers a blueprint on how fundamental care principles can be systemically integrated into common clinical instruments such as the EMRs to deliver medically reasonable goal-concordant care more reliably.^17^ Specific to our proposed process pertaining to establishing goals of care, we envision utilizing EMR software to create easily visible prompts or “pop-ups” to alert clinicians to consider the questions alluded to in Tables 1 and 2 in sequential manner. Furthermore, palliative care consultative teams could inform referring teams succinctly and more clearly that on initial visit, through prompts in their note template, that part of their consultative process will be a routine assessment of the patient and/or surrogate's understanding of the expected outcome from medical interventions.

A more transparent disclosure of their consultative process should foster trust with referring clinicians, as the expectation of what a palliative care consultation entails would be more clearly outlined. Therefore, referring clinicians may be less likely to conceptualize the process of establishing “goals of care” as a black box that is best if left to be performed by “the experts.” This also could translate into better success with attaining goal-concordant care, as both generalist and specialist providers may be more likely to participate in a simplified process that they can conceptualize and follow.

Measurement of goal-concordant care is important for maintaining accountability to high-quality care across health care systems. It may be facilitated through more standardized language and documentation of whether the patient's ultimate understanding of the primary aim of medical treatment aligned with what clinicians felt was medically possible (step 1) and whether the patient's individualized goal of medical treatment (step 2) was ultimately achieved.

## Abbreviations Used

AFHS: age-friendly health systems
EMRs: electronic medical records
HUC: health unit communicator
